# Effective Management of Giant Ventral Hernias: A Comprehensive Approach Combining Preoperative Botulinum Toxin Application, Modified Ramírez’s Component Separation, and Rives-Stoppa Hernioplasty

**DOI:** 10.7759/cureus.48967

**Published:** 2023-11-17

**Authors:** José Luis Serafio-Gómez, César Aragón-Quintana, Melanie Bustillos-Ponce, Omar Varela-Barraza, Beatriz Silva

**Affiliations:** 1 General Surgery, Chihuahua City General Hospital “Dr. Salvador Zubirán Anchondo”, Chihuahua, MEX; 2 General Medicine, Chihuahua City General Hospital “Dr. Salvador Zubirán Anchondo”, Chihuahua, MEX; 3 General Surgery, Hospital Regional de Alta Especialidad del Bajío, León, MEX

**Keywords:** hernia defect, incisional hernia, rives-stoppa hernioplasty, botulinum toxin, giant ventral hernia

## Abstract

Introduction

Giant ventral hernias are a surgical challenge due to their size and the need for a specialized approach during repair. Over the decades, abdominal wall surgery has evolved into a sophisticated field with a wide range of techniques aimed at improving patient outcomes. However, there is no universally accepted method suitable for repairing all giant ventral hernias. Surgeons must rely on a combination of techniques, choosing the approach that best matches their expertise, available resources, and the individual patient’s specific needs. This article explores the effective use of a combination of techniques, including preoperative botulinum toxin application, modified Ramírez’s component separation, and Rives-Stoppa hernioplasty, yielding excellent results and minimizing recurrences.

Objective

This study aims to provide a comprehensive literature review of giant ventral hernias. Additionally, we aim to share our experience in managing and repairing giant ventral hernias using a multi-modal approach, combining various surgical techniques with a focus on patient safety, reduced recurrence rates, and improved quality of life.

Methods

Between October 1, 2019, and October 1, 2021, six patients with giant ventral hernias were enrolled at our department of surgery. They received preoperative botulinum toxin A (BT) application, underwent corrective surgery involving modified component separation following the Ramírez method, and received Rives-Stoppa hernioplasty. Follow-up was conducted for at least six months.

Results

Six patients were included in the study: three women and three men. They had an average age of 53.6 years and an average body mass index of 31.8 kg/m^2^. The most common location of the hernia defect was supra and infraumbilical, among 66% of cases. The primary adverse effect associated with BT application was abdominal distension, reported in 33% of patients. No postoperative complications, such as abscesses or seromas, were observed. After the surgical procedure, the average hospital stay was 2.6 days, and no recurrences were noted within six months post-surgery.

Conclusion

The proposed method, which involves a combination of techniques, has demonstrated promising results based on our experience. However, to solidify these findings and better understand the full scope of this approach, further comprehensive statistical studies involving larger populations are essential. These studies will not only validate our results but also provide valuable insights for optimizing the management of giant ventral hernias.

## Introduction

Abdominal incisions often result in areas of inherent weakness. This area is often susceptible to dehiscence due to high intra-abdominal pressures, infectious complications, and alterations in wound healing. The formation of incisional hernias in up to 20% of cases is attributable to the failure of the proper closure of the abdominal wall as a consequence of the factors already described [1-4].

Correcting incisional hernias can be challenging, especially when there’s a loss of domain. Loss of domain occurs when the hernia defect grows to a size where the abdominal contents can’t be retained, leading to their protrusion into the hernial sac [5]. Giant ventral hernias, as defined by the European Hernia Society, encompass any ventral hernia greater than 10 cm, regardless of the presence of loss of domain [6].

The repair of giant ventral hernias carries a higher risk of postoperative morbidity compared to other hernia repair procedures. These hernias can impose a significant physical and psychological burden on patients, impacting their quality of life [7]. Achieving the best outcomes in giant ventral hernia repair involves an integrated approach that considers established principles and individual patient needs.

Despite the advancements in abdominal wall surgery over the last two decades, there is no universally standardized method for repairing giant ventral hernias. Various management approaches and component separation techniques have been described, each offering its own set of advantages and risks. In 1940, Goñi Moreno described the preoperative pneumoperitoneum, whose objective is to allow visceral reintroduction and its progressive adaptation to the abdominal cavity, reducing cardiorespiratory complications in the immediate postoperative period [1,2]. This allows relaxation due to the progressive distension of the musculature of the abdominal wall, which is retracted. It acts like the pneumoperitoneum in laparoscopy, facilitating the atraumatic dissection of adhesions. This innovative technique has proven effective in various surgical settings; however, we opted not to utilize it. This decision was based on practical considerations, including the specific characteristics of our patient cohort and logistical constraints. Additionally, patient cooperation, an essential factor for the success of this technique, was limited in our case. While the preoperative pneumoperitoneum has shown effectiveness in various surgical settings, its application was deemed impractical for the scope and context of our study. 

In recent years, botulinum toxin A (BT) has emerged as a complementary therapy frequently utilized in giant ventral hernia repair [8-10]. BT is a powerful neurotoxin derived from Clostridium botulinum, and when infiltrated into the abdominal wall muscles, it temporarily induces flaccid muscle paralysis by inhibiting the release of acetylcholine. The reduction in the tensile strength of muscle facilitates hernia repair. The maximum effect of BT is typically achieved two to four weeks after application and gradually diminishes [10-12]. Muscle function is usually recovered within three to six months after application. Over the past four decades, BT has evolved into a therapeutic tool for an expanding array of clinical applications, including dystonia, spasticity, achalasia, hyperhidrosis, bladder dysfunction, and pain management, among many others [13]. In the context of ventral hernia repair, muscle relaxation facilitates the apposition of defect edges without compromising the fascial integrity of the abdominal wall. This minimizes lateral muscle traction, subsequently reducing the need for manipulation and traction during surgical repair and decreasing the post-procedural analgesic requirement [11,12,14-17].

## Materials and methods

This study is a retrospective and descriptive analysis of a patient series from a second-level hospital in the northwest of Mexico. Patients diagnosed with giant incisional ventral hernias with or without loss of domain, from 2019 to 2021, were selected for analysis. The analysis encompassed a comprehensive review of the patients’ medical histories, preoperative and postoperative computed tomography scans, the preoperative application technique of botulinum toxin type A, adverse effects observed after its application, and an assessment of the benefits and complications experienced during the hernioplasty procedure performed. Additionally, patients were monitored for a minimum of six months after the surgical intervention.

Description of techniques

**Figure 1:**
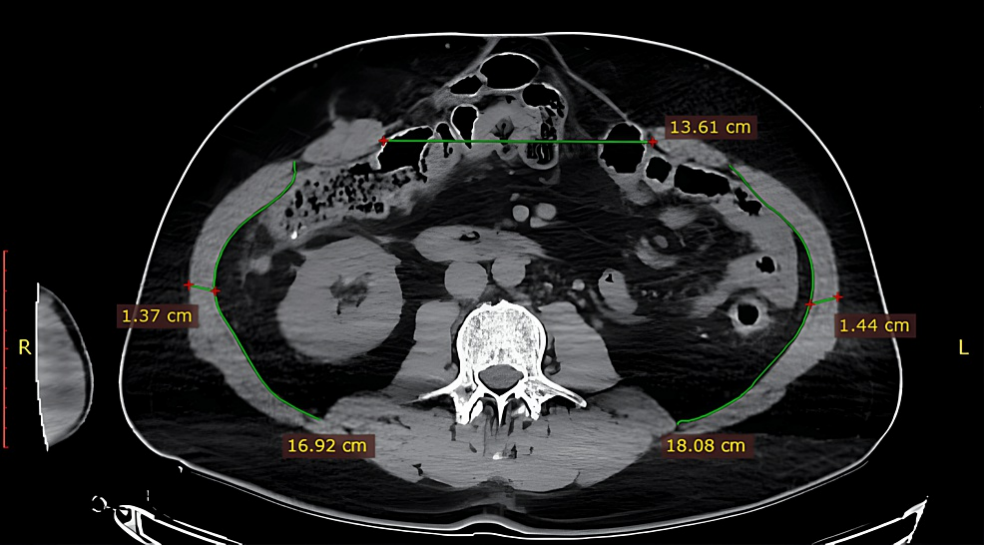
Preoperative computed tomography scan A simple computed tomography scan was conducted to determine information related to the hernia defects’ size, dimensions, and precise location

Before the application of BT, a simple plain computed tomography scan was performed to ascertain data regarding the size, diameter, and exact location of the hernia defects (Figure 1). Additionally, it offered insights into the characteristics of the ventral muscle blocks and delved into the depths of the abdominal wall muscles, ensuring a thorough preoperative assessment.

Application of botulinum toxin type A 

**Figure 2:**
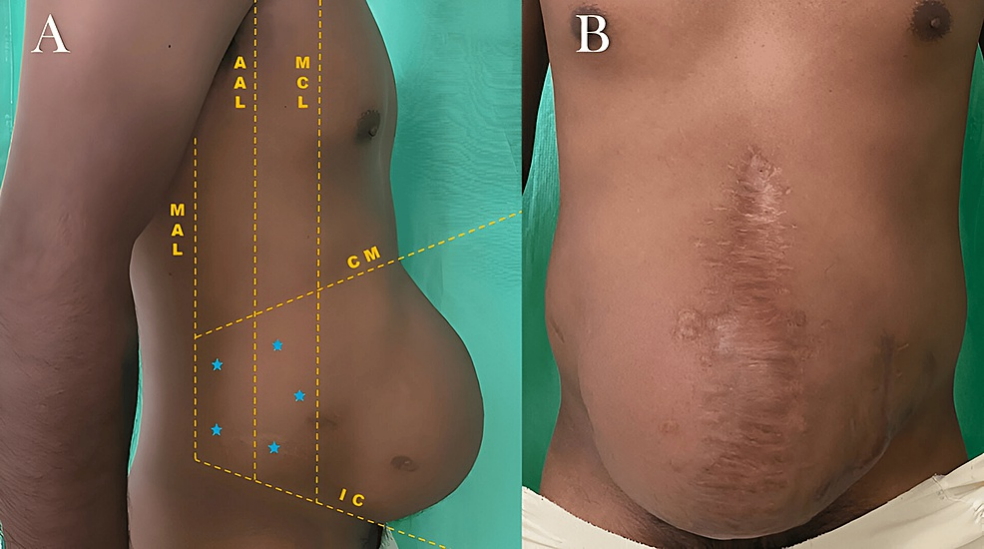
Application of botulinum toxin type A (A) Botulinum toxin A application sites (blue stars) (B) Preoperative view of giant ventral hernia four weeks after the application of botulinum toxin MAL, midaxillary line; AAL, anterior axillary line; MCL, midclavicular line; CM, costal margin; IC, iliac crest

Hematological and coagulation profile laboratory studies were requested to exclude any coagulopathy, which represents a relative contraindication for the application of botulinum toxin. Once the CT scans and laboratory tests were completed, the application sites were meticulously selected based on the points of maximal myoelectric stimulation of the abdominal wall, as proposed by Ibarra-Hurtado et al. [14]. These points were precisely located under ultrasonographic guidance and consisted of two points in the mid-axillary line, between the costal margin and the external iliac crest, and three points on the external oblique muscle border on each side (Figure 2A). A strict aseptic technique was rigorously maintained throughout the entire procedure. Plain 3% lidocaine was infiltrated into the subcutaneous space at the five marked points on each side of the aponeurotic defect. Using a 22G hypodermic needle (0.7 mm × 32 mm), 30 units of botulinum toxin (Botox®) were precisely injected at each of these points bilaterally in the internal and external oblique muscle, resulting in a total of 300 units applied per patient. Four weeks after the BT application, once the effects were fully evident, the hernioplasty procedure was performed (Figure 2).

Anatomic component separation technique by Ramírez

The Ramírez technique, described in 1990, was developed as a method to expand the abdominal cavity [8]. It involves the precise dissection of the abdominal wall between its various components. Conducting a bilateral, longitudinal dissection between both oblique muscles facilitates the liberation of the rectus abdominis muscle and the rotation of the posterior sheath, ultimately allowing for the reconstruction of the linea alba. The advantages suggested by this procedure center around utilizing innervated, vascularized, and autologous tissue to repair defects in the anterior abdominal wall. Beyond achieving closure without tension, the incorporation of these innervated myofascial flaps plays a role in replicating the dynamics of the original abdominal wall. In 1983, Ger and Duboys acknowledged the advantages of innervated contractile muscle over denervated fascia or synthetic mesh, emphasizing its capacity to better withstand stress and distribute tension more efficiently along the abdominal wall’s length [3]. These factors have contributed to a reduction in the average recurrence rate to approximately 10% [18].

Modified Rives-Stoppa technique

The Rives-Stoppa technique, originally described in 1980, involves the precise placement of a mesh within the preperitoneal and retromuscular space, where vascularization is optimal, followed by the primary closure of the anterior fascia [19]. This approach capitalizes on the advantageous intra-abdominal pressures, which facilitate the mesh’s integration into the surrounding tissues. By leveraging the very forces that contributed to hernia formation, it effectively prevents recurrences. Over time, this technique has become the standard for addressing complex incisional ventral hernias, resulting in remarkably low recurrence rates. Furthermore, its preperitoneal location ensures seamless compatibility with potential future abdominal surgeries [20].

Integrated approach: a combination of techniques for optimal results

**Figure 3:**
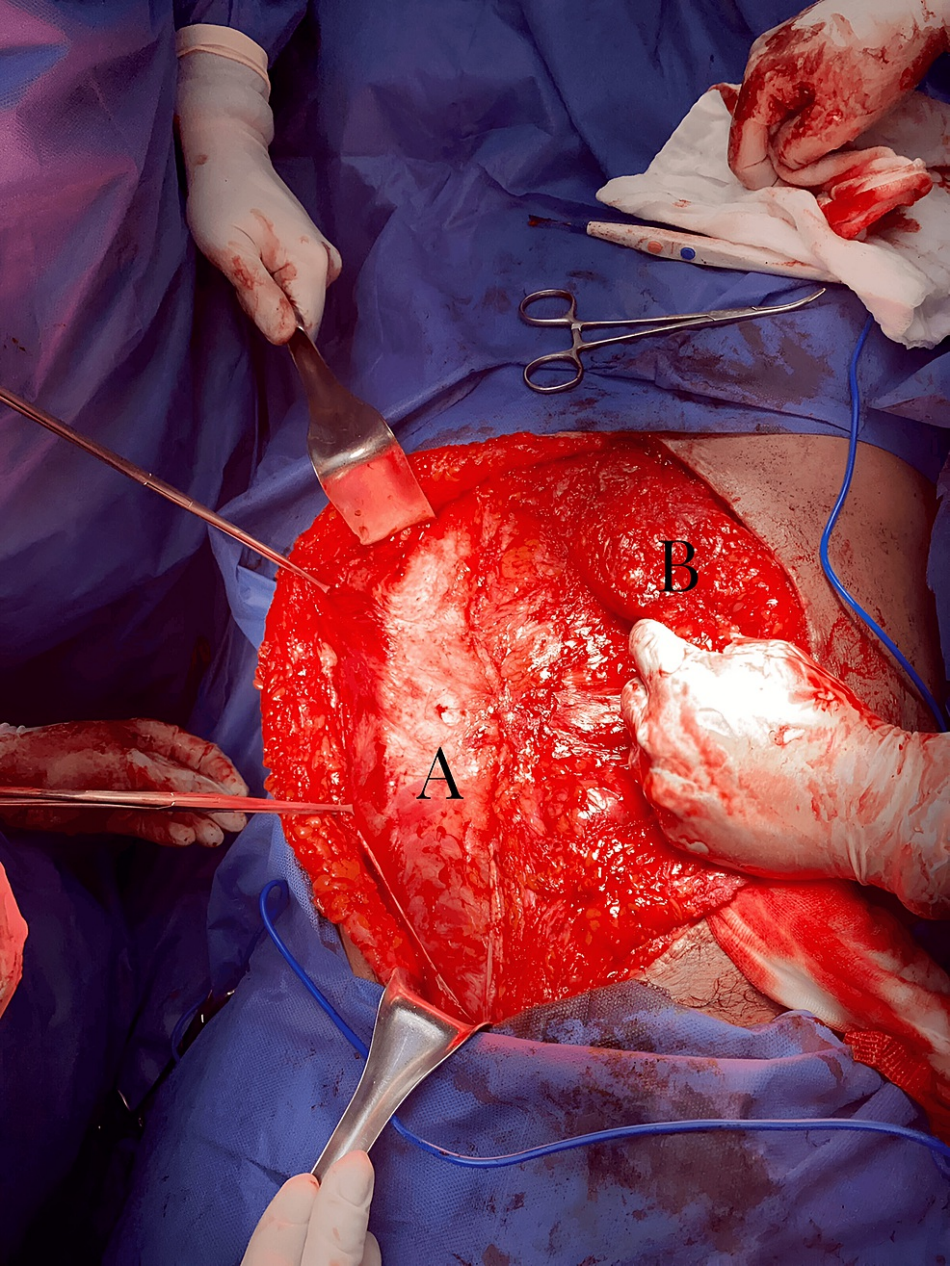
Anatomic component separation technique by Ramírez Anatomical component separation following the Ramírez method (A) Initiation of component separation using the Ramírez technique (B) Hernia defect

**Figure 4:**
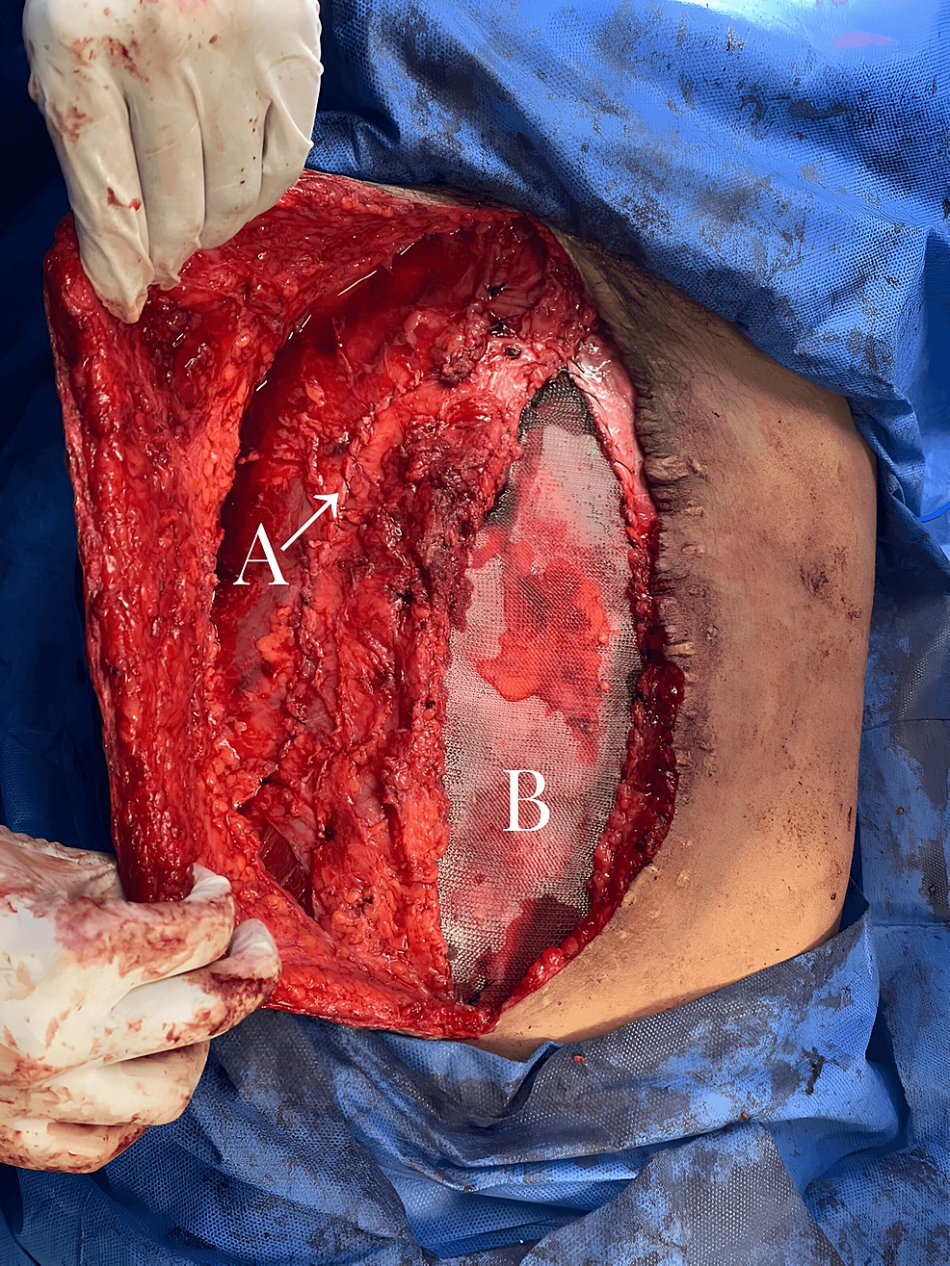
Combination of techniques In this photo, the reinsertion of the oblique muscles (A) and the placement of the retro-rectal mesh (B) can be observed after the combination of techniques and before the complete closure

The method performed on all patients involved a combination of three techniques already described and documented in the existing literature. This approach included the preoperative application of BT four weeks before the surgical event. The approach begins by removing the previous scar; then, the hernia defect is dissected. Subsequently, retrorectal dissection and component dissection are performed (Figure 3), starting with the oblique muscles and then proceeding with the rectus abdominal muscles. A medium-density macroporous polypropylene mesh is crafted and placed within the retromuscular of the rectus abdominis and preperitoneal space. Mesh fixation around its entire periphery and fascial closures were carried out with PDS® sutures (Figure 4). This is followed by the insertion of the oblique muscles and the anterior closure of the wall. Umbilical reconstruction was performed, and primary skin closure was achieved using PROLENE® sutures. Additionally, all patients were fitted with a closed drainage system equipped with an anti-reflux valve and a collection system.

The purpose and approach of this procedure evolved to the one described above. Initially, different techniques were applied separately in various patients, but the results were unfavorable. Therefore, the decision was made to combine these techniques, achieving a better outcome. This combination allowed for the closure of the wall without any tension, unlike what was observed when applying the techniques individually.

## Results

The study population and their demographic characteristics are outlined in Table 1, while information regarding the procedures conducted is presented in Table 2. All patients were referred to the general surgery outpatient clinic with a diagnosis of ventral hernia in the absence of complications. A total of six patients were included (three females and three males). The mean age was 53.6 years (range, 36-67 years) with a mean BMI of 31.8 (range, 22-41 kg/m^2^), and all patients had associated comorbidities, notably high blood pressure, which was present in all of them.

**Table 1:** Demographic characteristics of study participants BMI, body mass index; ASA, American Society of Anesthesiology Scale; HAS, systemic arterial hypertension; T2D, type 2 diabetes

Cases	Age (years)	Sex	Comorbidities	BMI (kg/m^2^)	ASA class	Previous surgical procedures
1	51	Male	HAS	31	2	Exploratory laparotomy
2	67	Female	Smoking, HAS	34	3	Eight previous hernioplasties
3	66	Female	T2D, HAS, morbid obesity, hypothyroidism, bipolar disorder	41	3	Cholecystectomy
4	36	Male	Smoking, HAS	22	2	Exploratory laparotomy + loop colostomy
5	55	Male	Smoking, HAS, T2D	33	3	Exploratory laparotomy
6	47	Female	HAS	30	2	Exploratory laparotomy

**Table 2:** Preoperative and postoperative aspects of the population EHS, European Hernia Society; IU, infraumbilical; EG: epigastric; F, flank; BT, botulinum toxin A

Cases	Evolution (months)	Hernia defect location	EHS incisional hernia classification	Post-BT complications	Transverse diameter of hernia defect before BT (cm)	Transverse diameter measured intraoperatively (cm)	Loss of domain	Hospital stay (days)	Follow-up (months)	Postoperative complications
1	24	IU	M4 W3	-	15.9	11.6	No	3	11	-
2	6	EG	M2 W3	-	13.6	9.5	No	2	16	Wound infection + dehiscence
3	7	F	L2 W3	-	13.8	8.5	Yes	3	6	-
4	6	IU	M4 W3	-	15.2	12.7	Yes	2	12	-
5	9	IU	M4 W3	Abdominal distension + cough	21.7	17.3	Yes	3	20	-
6	7	IU	M4 W3	-	13.9	8.9	No	3	9	-

**Figure 5:**
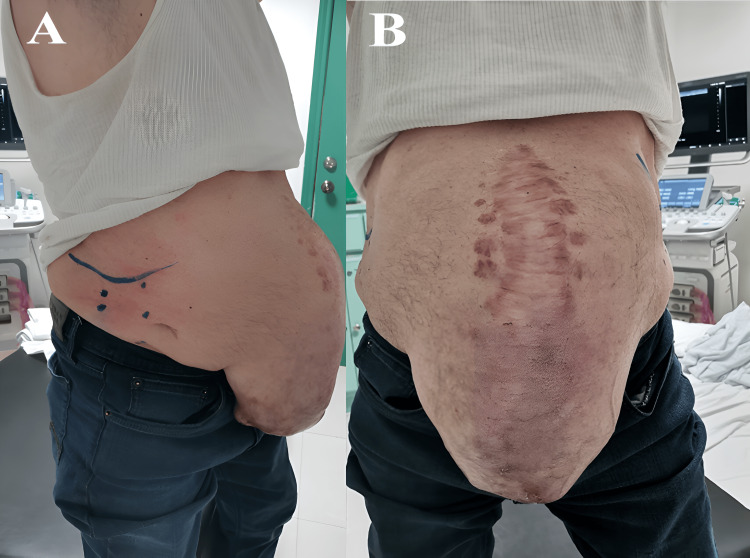
Giant ventral hernia in a 55-year-old male (A, B) The patient experienced lower back pain, respiratory challenges, limited mobility, and aesthetic concerns

At the initial consultation, the main reason for seeking medical attention was primarily driven by cosmetic and aesthetic concerns. However, it’s noteworthy that all patients experienced symptoms of discomfort and limited mobility. Notably, none of them reported abdominal pain as their main complaint. One patient presented lumbalgia and respiratory difficulties, particularly when lying in a supine position, which prompted their visit (Figure 5).

Every patient had a history of prior abdominal surgical interventions, with exploratory laparotomy being the most common, representing 66% of cases. As for the hernia defect, its most prevalent location was in the midline (both supra- and infraumbilical) in 66% of cases, one supraumbilical (16%), and one on the right flank (16%). The mean transverse diameter of the hernia defect was 15.6 cm, with 50% of the patients exhibiting a loss of domain, indicated by a Tanaka index greater than 20%. Only two patients had a history of previous hernioplasties, having undergone eight procedures, with the last one performed 13 months before the start of their current treatment.

All patients received BT according to the previously described technique, and only two of them displayed additional symptoms following the administration. Abdominal distension was the most frequent, affecting one-third of the patients, while only one patient reported the onset of cough after BT administration (16.7%). The rest of the patients did not report any symptoms associated with the application.

**Figure 6:**
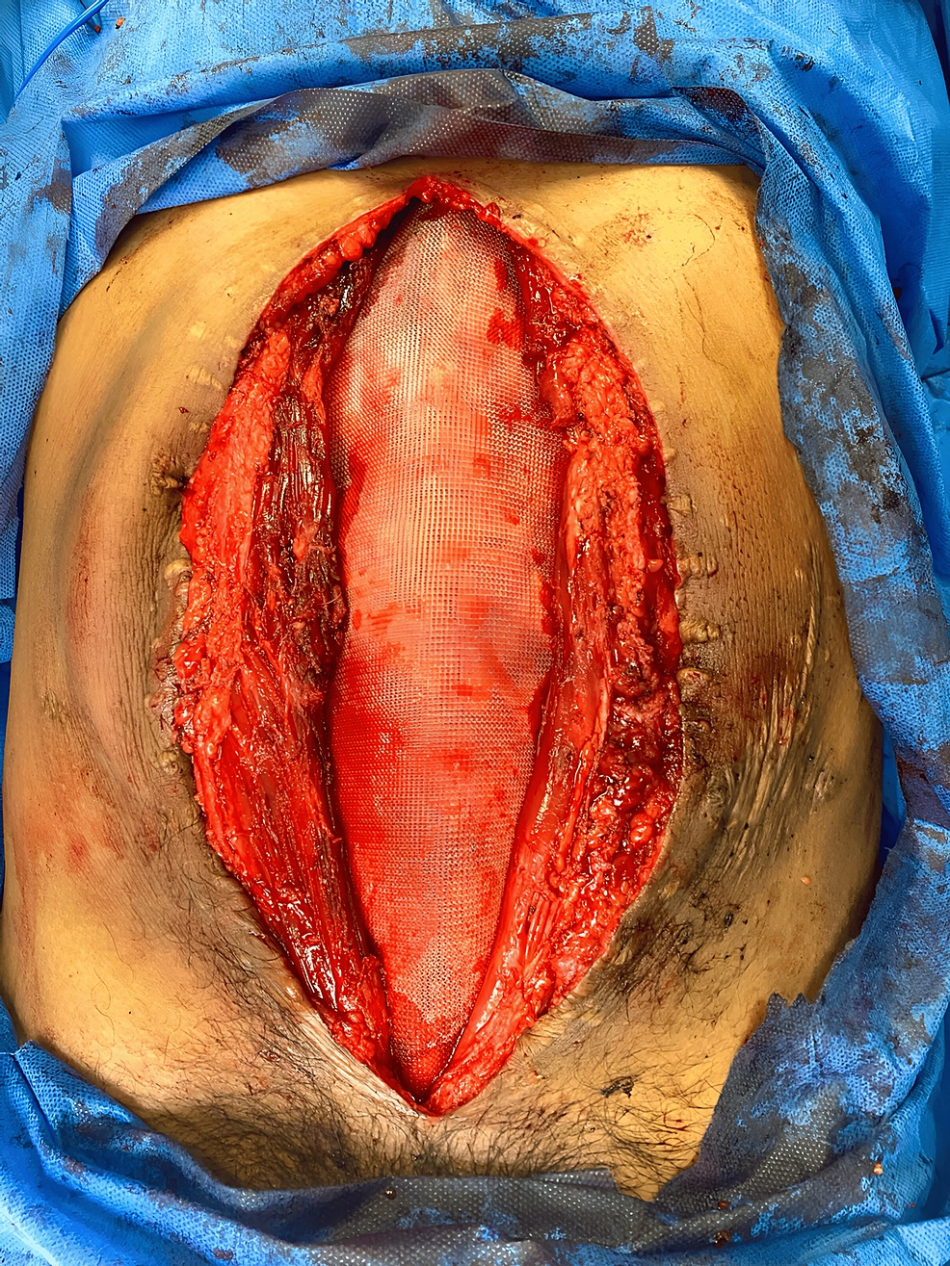
Modified Rives-Stoppa technique Rives-Stoppa hernioplasty, with the precise placement of a mesh within the preperitoneal and retromuscular space

In all cases, component separation was performed using the Ramírez technique. This was combined with Rives-Stoppa hernioplasty featuring preperitoneal mesh modifications already described (Figure 6). No intraoperative complications were encountered. The average length of hospital stay was 2.6 days. However, one patient experienced a surgical site complication (16.7%) in the early postoperative period. This complication manifested six days after the surgical procedure and was characterized by persistent abdominal pain, abdominal distension, wound dehiscence, and purulent discharge. As a result, a second surgical intervention was required to drain a subcutaneous abscess, followed by daily wound cleansing until skin closure was achieved.

**Figure 7:**
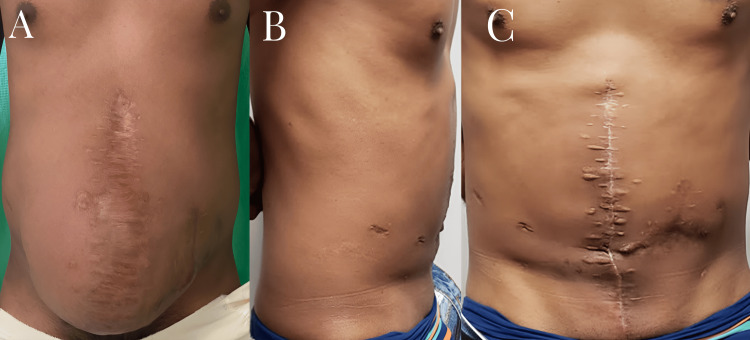
Preoperative and postoperative images of a giant ventral hernia (A) Preoperative view of giant ventral hernia (B, C) Postoperative view of the same patient three weeks after surgical procedure

The long-term follow-up had an average duration of 12.3 months. A minimum follow-up period of six months was established for all participants. Throughout this time, there were no instances of hernia recurrences or additional complications observed (Figure 7).

## Discussion

Clinical experience with BT in the treatment of ventral hernias has extended over a decade. This has provided a comprehensive understanding of its fundamental aspects. However, there is still a lack of standardization in the technique and its indications for different populations. This limitation is due to the significant variability in the methodology of current studies. While these studies have qualitatively and quantitatively demonstrated the clinical significance of using BT in ventral hernia repair, they have also unveiled a wide range of potential interventions for addressing this common issue.

Systemic arterial hypertension was the most prevalent comorbidity in the entire study population. It was closely followed by obesity. The average BMI was 31.8 kg/m^2^, with 83% of the study population having a BMI greater than 30 kg/m^2^ and 16.7% having a BMI exceeding 40%. These findings align with results from other studies in which obesity was the most common comorbidity among patients [21].

In this study, a preoperative dose of Botox® (300 units) was administered to six patients with giant ventral hernias, resulting in the reduction of hernia defects in all cases. The correction of the hernias was achieved through a combined approach of techniques. This approach involved the preoperative application of BT, a modified component separation technique based on the Ramírez method, and modified Rives-Stoppa hernioplasty.

The reduction in hernia defect size became evident following the application of 300 units of BT, with intraoperative measurements showing a decrease of 2-4 cm. Notably, previous studies have demonstrated significant outcomes. For instance, using 300 units resulted in a reduction of up to 58% in hernia defect diameter and an increase of up to 4 cm in bilateral muscle length [15]. Similarly, a dosage of 500 units led to a reduction of up to 4.79 cm in hernia defect size and an increase of up to 2.6 cm in bilateral muscle length [22]. Furthermore, the application of 100 units of BT resulted in a reduction of up to 50% in the total hernia sac area and a decrease in tension during the surgical repair process [18,23].

In this study, tomographic evidence indicated improved abdominal compliance following BT application. This was attributed to a decrease in content moving into the abdominal cavity due to the flaccid paralysis of the lateral abdominal muscles. This facilitated the closure of musculature, especially in the cases of giant and complex hernias [17].

The most common adverse effect post-BT application was abdominal distension (33%), as reported in other studies. Particularly, one patient (16.7%) developed a cough several days after BT application. This cough could be linked to the importance of abdominal muscles on respiration, a phenomenon observed in two other studies. This underscores the importance of exercising caution when administering BT to patients with chronic respiratory conditions [15,23].

Component separation was performed in all patients, potentially leading to an overestimation of the BT effect. However, only one study recommends a hernia transverse diameter greater than 18 cm as an indication of component separation [15,21]. 

The recurrence rate, after an average 12.3-month follow-up, was 0%. This aligns with results seen in studies with similar recurrence rates, even in follow-ups lasting up to 49 months [18,22,24]. However, this differs from other studies reporting a 9% recurrence rate [25].

Surgical site infection was the most common complication related to the surgical procedure, occurring in 16.7% of cases in this study. Similar incidence rates, ranging from 28% to 40%, have been reported in other studies [18,22,23,25]. However, the administration of BT did not increase the incidence of surgical site infections. It’s important to consider that the patient experiencing this complication had underlying risk factors, including type 2 diabetes and hypothyroidism.

Lastly, several studies have reported the effectiveness of BT in decreasing the requirement for postoperative analgesia. This effect extends until the toxin’s activity at the neuromuscular junction subsides, leading to improved patient clinical outcomes and reduced consumption of opioids or postoperative analgesia [25,26].

Limitations of the study

The study encountered several limitations that warrant consideration. Firstly, the relatively small sample size and the restricted six-month follow-up duration may impede the ability to definitively ascertain the long-term efficacy and potential complications associated with the combined techniques. Moreover, the focus on patients amenable to these techniques introduces a potential selection bias, limiting the generalizability of the findings to the broader population of giant ventral hernia patients. Being a single-center study, the results might not fully capture variations present in different healthcare settings and patient populations. Additionally, the absence of a comparative group receiving alternative treatments hinders the establishment of the combined approach’s superiority or inferiority. The short-term follow-up of six months, while providing initial insights, falls short in assessing the durability of repairs and identifying potential late complications. Recognizing and addressing these limitations will contribute to a more comprehensive and nuanced interpretation of the study’s outcomes.

## Conclusions

Giant ventral hernias present a complex surgical dilemma due to their size and the specialized approach needed for their treatment. Since there is no universally accepted technique for repairing all giant ventral hernias, surgeons have the flexibility to use a combination of the described techniques tailored to their expertise, available resources, and patient-specific considerations. The combination of techniques described in this article offers a viable therapeutic option for treating giant ventral hernias. These techniques include preoperative BT application, modified component separation based on the Ramírez method, and hernioplasty using a Rives-Stoppa mesh. This approach has shown minimal adverse effects and complications, ensuring a tension-free fascial closure and reducing the risk of post-surgical recurrences. To draw more meaningful statistical comparisons with alternative techniques or combinations, it’s essential to conduct additional clinical trials involving larger populations.
